# Supplementary material for: Effect of Heavy Ion 12C6+ Radiation on Lipid Constitution in the Rat Brain
Source: Molecules. 2020 Aug 18;25(16):3762. doi: 10.3390/molecules25163762 (PMC7465761; doi:10.3390/molecules25163762)
Supplement: Supplementary file 1 [file molecules-25-03762-s001.pdf]

Supplementary Information

Effect of Heavy Ion $^{12}C^{6+}$ Radiation on Lipid Constitution in the Rat Brain

Bo Li[1,2]*, Chu Han[2,3], Yuanyuan Liu[2,3], Nafissa Ismail[4,5], Kevin Smith[4], Peng Zhang[1], Zixuan Chen[1] , Rongji Dai[1]* and Yulin Deng[1]*

[1] Beijing Key Laboratory for Separation and Analysis in Biomedicine and Pharmaceuticals, School of Life Science, Beijing Institute of Technology, Beijing, 100081, China

[2] Advanced Research Institute of Multidisciplinary Science, Beijing Institute of Technology, Beijing, 100081, China.

[3] School of Chemistry and Chemical Engineering, Beijing Institute of Technology, Beijing, 100081, China.

[4] Neuroimmunology, Stress and Endocrinology (NISE) Lab, School of Psychology, Faculty of Social Science, University of Ottawa, Ottawa, ON, Canada.

[5] Brain and Mind Research Institute, University of Ottawa, Ottawa, ON, Canada.

Table S1. Rat body weight (in grams) before and after carbon heavy ion irradiation. (*p<0.05, n=8)

	Subject	Starting (g)	Day of Irradiation (g)	7 Days Post-Irradiation (g)
**Control Group**	C1	169	186	214
	C2	189	211	244
	C3	178	191	219
	C4	175	200	235
	C5	185	211	247
	C6	178	202	231
	C7	163	188	205
	C8	169	187	207
	Average±SD	175.75 ± 8.66	197 ± 10.45	225.25 ± 16.31
**Irradiation Group**	M1	177	204	213
	M2	179	218	220
	M3	167	192	184
	M4	164	193	202
	M5	162	197	191
	M6	181	210	222
	M7	174	186	205
	M8	176	187	205
	Average±SD	172.50 ± 7.19	198.38 ± 11.38	205.25 ± 13.22 *

Table S2. Lipid classification of untargeted lipidomics in the rat brain.

Lipid Category	Subclass	Total Number	Differential Lipid Number
Fatty Acyls （FA）	Acyl Carnitines（ACar）	40	
	Fatty acid hydroxy fatty acid （FAHFA）	60	1
	Fatty esters（FA）	50	
Glycerolipids （GL）	Monoacylglycerol（MAG）	25	
	Diacylglycerol（DAG）	469	13
	Triacylglycerol（TAG）	348	6
	Pentadecylbenzylphosphonic acid （BMP）	14	
Glycerophospholipids （GP）	Lysophosphatidylcholine（LPC）	139	1
	phosphatidylcholine（PC）	182	1
	Phosphatidylethanolamine（PE）	341	2
	Lysophosphatidylethanolamine （LPE）	202	2
	Phosphatidylglycerol（PG）	168	
	Phosphatidylinositol（PI）	207	
	Phosphatidylserine（PS）	103	
	Phosphatidic acid（PA）	57	
	Lysophosphatidylglycerol（LPG）	28	
	Lysophosphatidylinositol（LPI）	18	
	Lysophosphatidylserine（LPS）	1	
	Diacylglycerophosphates（PMeOH）	20	
Sterol Lipids （ST）	Cholesterol ester（CE）	64	
Sphingolipids （SP）	Sphingosine	6	
	Sphinganine	4	
	Phytosphingosine	5	
	Sphingomyelin（SM）	479	3
others		1	

Table S3. Untargeted lipidomics of the rat brain in control group and irradiation group.

Average Rt(min)	Average Mz	Metabolite name	Control group			Irradiation group			VIP(C vs M)	FC(C vs M)	P(C vs M)
			C1	C2	C3	M1	M2	M3			
2.605	522.417	DAG 28:4	117284	245911	69784	317065	265473	430045	1.898	0.428	0.0271
9.596	690.545	DAG 42:11e	65196	88851	65030	349903	159679	127513	1.369	0.344	0.0584
9.088	703.583	TAG 39:0	308157	500037	747014	347891	195620	263817	2.449	1.926	0.0686
9.208	730.605	TAG 42:5	252808	292721	676732	210205	81230	96689	2.732	3.149	0.0601
8.445	741.561	SM t36:4	23643	197774	384214	424761	437169	278936	1.753	0.531	0.0992
9.237	747.611	SM t36:1	243788	403693	708463	75216	77530	189946	3.319	3.957	0.0377
8.805	748.623	LPE 37:0	106360	207751	279624	350459	281458	369641	1.336	0.593	0.0377
9.271	754.604	TAG 44:7	123100	111334	255100	64031	31960	60075	1.092	3.137	0.0390
9.867	759.643	TAG 43:0	53260	211016	380066	41699	29947	57030	1.689	5.007	0.0718
10.342	766.672	DAG 47:8e	85130	200786	307124	67214	90958	96996	1.107	2.324	0.0785
10.897	768.742	DAG 45:0	681359	614780	296489	308989	203471	93057	3.233	2.630	0.0351
8.536	775.588	TAG 45:6	189408	171051	2294	178002	322652	271177	1.340	0.470	0.0677
8.29	782.656	DAG 47:7	383800	292612	86856	78292	124958	101870	1.501	2.502	0.0804
11.017	782.759	DAG 46:0	550878	378929	322127	76219	97455	60530	3.333	5.345	0.0041
10.985	794.757	DAG 47:1	1222140	774987	635904	210821	444946	225621	5.737	2.987	0.0193
11.117	796.779	DAG 47:0	535792	669545	260778	216248	400566	102193	2.447	2.039	0.0844
11.204	810.794	DAG 48:0	216548	295842	233060	150391	157421	129630	1.009	1.704	0.0079
10.155	812.749	DAG 50:6e	42558	734241	664413	59621	147444	99503	3.716	4.701	0.0813

11.041	820.776	DAG 49:2	383931	240334	183229	224101	169547	57875	1.166	1.788	0.0995
10	851.717	TAG 50:3	38852	49675	85711	128291	188463	167292	1.015	0.360	0.0051
7.469	878.610	PE 48:12e	720584	899684	743253	1254336	849509	1995552	5.685	0.577	0.0821
11.551	878.853	DAG 53:1	408558	323845	192489	249949	168493	177704	1.077	1.551	0.0910
11.679	880.876	DAG 53:0	114865	265012	222177	43270	92422	44105	1.383	3.349	0.0207
6.738	945.677	SM d51:10	134843	355385	266013	98279	103272	106655	1.467	2.454	0.0401
8.293	1194.888	PE 70:15	190352	385004	439817	151471	119959	164812	1.896	2.327	0.0330
2.683	478.294	LPE 18:1	697751	552818	992497	375393	422413	521059	3.027	1.701	0.0433
8.43	487.383	FAHFA 31:4	196894	252992	199168	125461	145924	65448	1.023	1.927	0.0132
2.297	540.331	LPC 16:0	190031	134515	113928	313992	173979	327156	1.234	0.538	0.0404
8.564	778.563	PC 32:0	143084	120433	89208	296622	310891	162121	1.365	0.458	0.0248

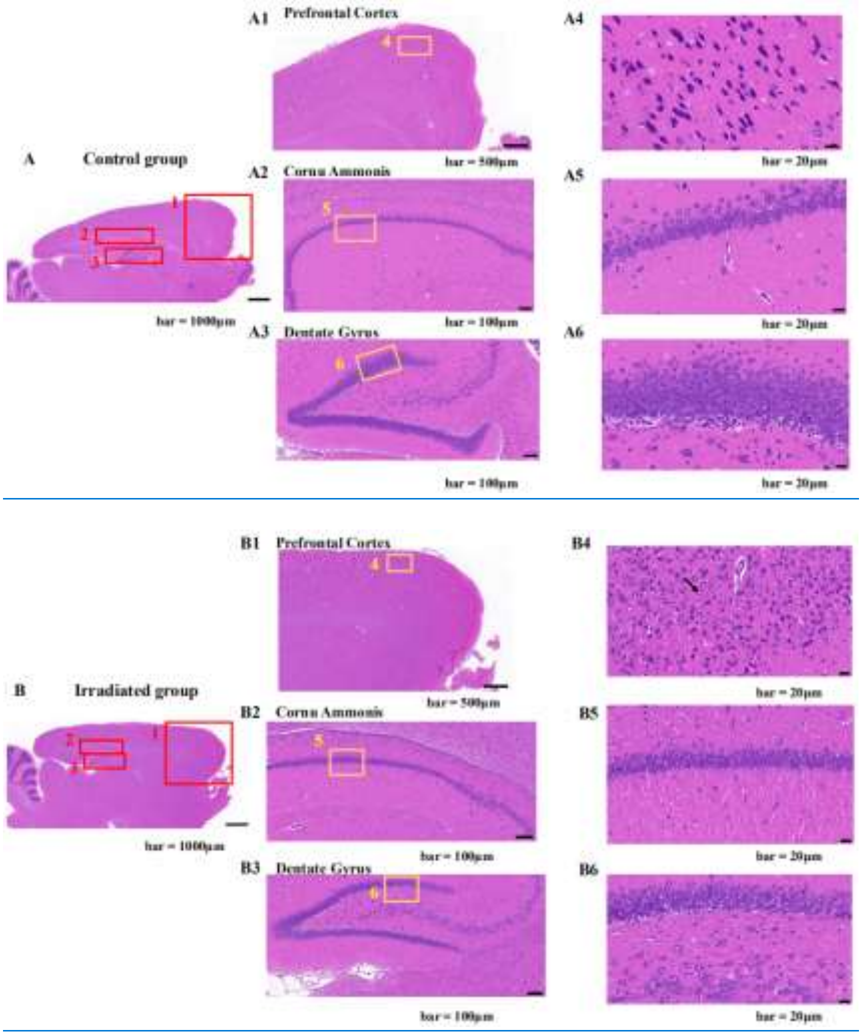

Figure S1. H&E staining of rat neuronal cell morphology in the prefrontal cortex and hippocampus (n=1) following sham-irradiation (**A**) or irradiation (**B**). Arrow indicates cells which have been altered due to irradiation.

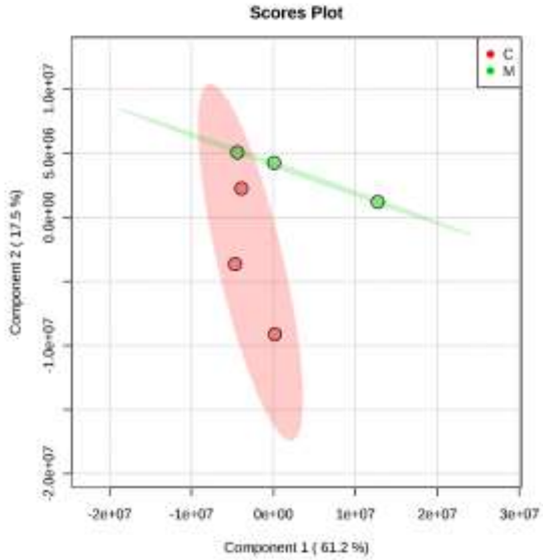

Figure S2. PLS-DA plot visualizing brain-tissue lipidomics profiles from sham-irradiated rats (C) and irradiated rats (M) after 7 days.

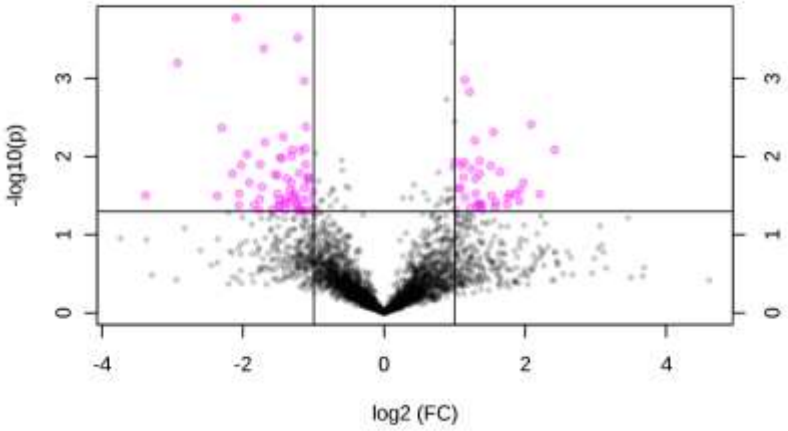

Figure S3. Volcano plot data from univariate analysis showing fold change in metabolites (black = non-significant, pink = significant) Pink dot represents significant metabolites.

Deleted:

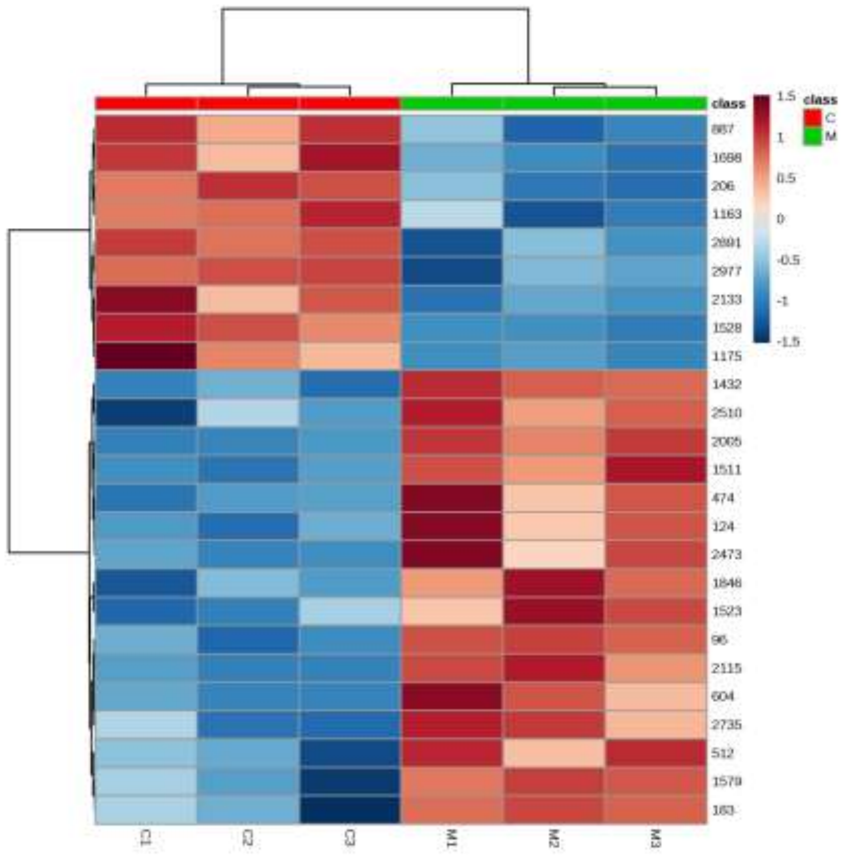

Figure S4. Hierarchical cluster analysis (HCA) of control (C) and irradiation (M) group metabolism. Color intensity indicate mean metabolic ratio increases (red) decreases (blue)
